# Magnitude and correlates of cognitive impairment among major depressive disorder patients in Addis Ababa: institution based cross-sectional study

**DOI:** 10.1186/s13104-019-4184-5

**Published:** 2019-03-14

**Authors:** Abebe Ambaw, Getachew Tesfaw Desalegn

**Affiliations:** 1 Jugol Referral Hospital, Harra, Ethiopia; 2 Department of Psychiatry, College of Medicine and Health Sciences, University of Gondar, Gondar, Ethiopia

**Keywords:** Cognitive impairment, Major depressive disorder, Mini Mental Status Examination

## Abstract

**Objective:**

Cognitive impairments are now widely recognized and emerging as an important therapeutic target in a patient with a major depressive disorder (MDD). It associated with a more deteriorating course of illness among MDD patients. Therefore, understanding the level of cognitive impairment and associated factors is crucial to provide optimal care for MDD patients.

**Result:**

The proportion of cognitive impairment among MDD patients was found to be 54.4% (95%, CI (49.6, 59.3). Factors significantly associated with having cognitive impairment were age adjusted odds ratio (AOR) = 3.00, 95%, confidence interval (CI): (1.49, 6.03), educational status, (AOR = 5.36, 95% CI 2.16, 13.26), employment status (AOR = 3.63, 95 CI 1.99, 6.62), duration of the illness (AOR = 3.16, 95% CI 1.31, 7.64), having co-morbid psychiatric illnesses (AOR = 2.16, 95% CI 1.26, 3.71), and illness relapse (AOR = 2.97, 95% CI 1.54, 5.73).

**Electronic supplementary material:**

The online version of this article (10.1186/s13104-019-4184-5) contains supplementary material, which is available to authorized users.

## Introduction

Major depressive disorder (MDD) is one of the most common mental illnesses with an estimated lifetime prevalence of 16.6%. More than 350 million people estimated to have MDDs globally [1].

The World Health Organization (WHO) Global Burden of Disease study ranked that depression is the most serious disorder in the world for total disability-adjusted years among people in the middle years of life [2]. Major depressive disorders can negatively affect cognitive function and cognitive loss after the onset of illness [3, 4].

Cognitive impairment (CI) is when a person has trouble concentrating, remembering, learning new things, or making decision that affect every day life [5]. And different factors could play a role in the development of CI secondary to depression. These include length of the depression, being female, the number of hospitalizations, education, duration of illness, and genetic [6–8].

Cognitive dysfunction is a core component of MDD [9–12]. But its prevalence is high inpatient setting than outpatient [13]. Almost half of the MDD patients have been affected by CI [14].

In MDD, the most commonly impaired cognitive domains are attention, memory, executive function, and it had been estimated to occur in about two-thirds of depressed patients [15, 16]. Executive and attention dysfunctions typical features in MDD patients [17]. And its functional impairment has been started from the first episode to recurrent episode [7, 17, 18]. Which in turn difficulty in concentration, and impaired decision making during actual patients’ examination [17, 19].

Although it was first thought that CI in MDD was state-related, there was increasing evidence suggesting that its abnormalities persist beyond depressive episode [20]. Different studies showed that adults experiencing their first major depressive episode performed were significantly high CI such as attention, visual memory, verbal fluency, and cognitive flexibility, with small to moderate effect sizes [21, 22]. Between one-third and one-half of remitted depressed patients were thought to be affected by cognitive dysfunction [16]. It is a common feature of MDD, contributing to the serious decline in patients’ quality of life difficult to concentrate, to make decisions, and “forget everything” [5, 23].

Persistent cognitive dysfunction influences therapeutic compliance, coping capacities, and therapeutic relationship [24]. Recurrent depression had been worse cognitive performance and executive function [25, 26]. Cognitive dysfunction in MDD is a relevant contributing factor inpatient clinical and functional outcomes [27]. The management of CI remains an uncover need in the treatment of MDD [28]. And it is associated with MDD. Therefore, it is becoming a key target of treatment in its own right and future studies [29].

Although this area of research is still emerging, it is important to consider these problem as possible treatment targets and growing evidence of significant impairment in cognitive functioning in MDD [30]. The emphasis on MDD collectively has left a gap in the research [31].

However, to the best of our knowledge, there is no published study done on CI and associated factors among MDD in Africa and Ethiopia. This study is therefore aimed to assess the prevalence and associated factors of CI among MDD patients at AMSH with a view of informing the development of the appropriate interventions.

## Main text

### Methods

An institution-based cross-sectional study was conducted at AMSH from May to July 2017, Addis Ababa Ethiopia. It is the only mental health hospital in Ethiopia and has a total of 300 beds. Currently, 1746 MDD patients are monthly on follow up.

All patients with MDD who were on follow up at AMSH at the outpatient department who were available during data collection period. Those MDD patients critically ill were excluded from the study. Sample size was calculated by using the single population proportion formula involving the using Epi-info version 7 with a 95% CI, 5% margin of error and the total of 421 MDD patients were recruited randomly by using systematic sampling technique.

Data were collected using a pre-tested interviewer-administered questionnaire, which contains CI as the dependent variable and other several explanatory variables.

The Mini-Mental Status Examination was used to measure CI among MDD patients and young adults whose age range from 18 to 85 years [32, 33].

Cognitive impairment was measured by using by MMSE test with the cutoff points ≤ had CI [34]. Social support was assessed by using Oslo 3-item social support scale and the sum score scale ranges from 3 to 14 with three broad categories: “Poor support” 3–8, “moderate support” 9–11 and “strong support” 12–14, respectively [35].

Data were entered to Ep-data software after checking completeness and imported SPSS version 21 for analysis. Bi-variable and multivariable logistic regression analysis was done to see the association of each independent variable with the outcome variable. Those variables having a p-value less than 0.2 were entered into the multivariate logistic regression model to identify the effect of each independent variable with the outcome variable. A p-value of less than 0.05 was considered statistically significant and AOR with 95% CI was calculated to determine association. We followed the methods of the previous study was done in Ethiopia [36].

### Results

#### Socio-demographic characteristics

From a total of 421 participants recruited for this study 395 completed the survey making a response rate 93.3%. The mean age of the participants was 37.2 (± 10.72) years. About half of respondents (50.4%) were females and 192 (48.6%) were married. Around one-third (28.9%) attended primary school, Orthodox religion followers accounted for 48.4% and 240 (60.8%) were currently working (Table 1).

**Table 1:** Distribution of MDD patients by demographic factors among MDD patients at AMSH, Addis Ababa, Ethiopia 2017 (n = 395)

Variable	Frequency	Percent
Sex		
Male	196	49.6
Female	199	50.4
Age		
18–25	102	25.8
26–40	150	38.0
41–59	143	36.2
Religions		
Orthodox	191	48.4
Muslim	143	36.2
Others^a^	61	15.4
Marital status		
Single	163	41.3
Married	192	48.6
Others^b^	40	10.1
Educational status		
Unable to read and write	94	23.8
Primary	114	28.9
Secondary	108	27.3
Tertiary	79	20
Occupational status		
Currently working	240	60.8
Not working	155	39.2

^a^Protestant, catholic and adventist

^b^Widowed, separated and divorced

#### Clinical and psychosocial characteristics

Among the participants, 274 (69.4%) developed MDD after the age of 25 years and three-forth 75.2% had been with the illness for more than 10 years. About half (50%) of the respondents had co-morbid psychiatric disorders and 139 (35.2%) of them had been hospitalized for MDDs (Table 2).

**Table 2:** Distribution of clinical and psychosocial factors among MDD patients at AMSH Addis Ababa, Ethiopia 2017 (n = 395)

Variable	Frequency	Percent
Co-morbidity		
Yes	198	50.1
No	197	49.9
Drug		
Antidepressant	217	54.9
Antidepressant, antipsychotic and others	178	45.1
Frequency of medication		
One time per day	226	57.2
Two times per day and three times per day	169	42.8
Duration of the illness (years)		
< 5	43	10.9
5–10	55	13.9
> 10	297	75.7
First illness (in the year)		
< 25	121	30.6
> 25	274	69.4
Treatment duration (years)		
< 6	242	61.3
> 6	153	38.7
Relapse		
Yes	172	43.5
No	223	56.5
Previous hospitalization		
Yes	139	35.2
No	256	64.8
Social support		
Strong	51	12.9
Moderate	166	42.0
Poor	178	45.1

#### Substance-related factors

Regarding substance use 31.9% and 28.9% of the participants were lifetime and currently substance users (Additional file 1).

#### Prevalence of cognitive impairment

This study showed that the prevalence of CI among study participants was found to be 54.4% with 95% CI (49.6, 59.3).

#### Factors associated with cognitive impairment

Among variables, sex, age, education, and occupational status, having co-morbid psychiatric illnesses, duration of treatment, previous hospitalization, relapse and duration of the illness were found to have a p-value less than 0.2 from bi-variable logistic regression and were considered for the multivariable logistic regression model.

A multivariate analysis suggest that, participants who did not read and write were 5.4 times more likely to have CI as compared to patients who achieved college and above level of education (95% CI 2.16, 13.26). Respondents who were not working 3.63 times more likely to develop CI than who were working (95% CI: 1.99, 6.62).

Participants whose age ranges between 41 and 59 years were increased risk of CI by three times (95% CI 1.49, 6.03) as compared to whose age ranges between 18 and 25 years. Furthermore, co-morbid psychiatric disorders two times increased CI as compared to no psychiatry co-morbid illnesses (95% CI 1.26–3.59). Similarly, respondents who had longer duration of treatment 3.16 times more likely to have CI than who had short duration of treatments (95% CI 1.31–7.64). Finally, having relapse were increased CI by 3.13 times (95%, CI (1.57–6.25) as compared to who did not have relapse history. On the other hand, sex, previous hospitalization and treatment duration had not associated with cognitive impairment (Table 3).

**Table 3:** Factors associated with cognitive impairment among MDD patients at AMSH, Addis Ababa, Ethiopia (n = 395)

Variables	Categories	Cognitive impairment		COR, CI (95%)	AOR, CI (95%)
		No	Yes		
Sex	Male	104	92	1	1
	Female	76	123	1.83 (1.23, 2.73)	1.65 (0.93, 2.93)
Age	18–25	67	35	1	1
	26–40	74	76	1.96 (1.17, 3.30)	1.43 (0.73, 2.79)
	41–59	39	104	5.11 (3.87, 12.57)	3:00 (1.49, 6.03)***
Presence of occupation	Yes	142	98	1	1
	No	38	117	4.46 (2.85, 6.98)	3.13 (1.76, 5.59)***
Education	Can’t read and write	14	80	7.56 (3.68, 15.56)	5.92 (2.45, 14.28)***
	1–8 grade	51	63	1.64 (0.92, 2.92)	1.49 (0.72, 3.13)
	9–12 grade	70	38	0.72 (0.39, 1.30)	0.68 (0.34, 1.48)
	Diploma and above	45	34	1	1
Co-morbidity	Yes	71	127	2.22 (1.48, 3.32)	2.16 (1.26, 3.71)*
	No	109	88	1	1
Treatment duration (years)	< 6	128	114	1	1
	< 6	52	101	2.18 (1.43, 3.32)	1.53 (0.69, 3.39)
Relapse history	Yes	56	116	2.59 (1.72, 3.93)	2.97 (1.54, 5.73)**
	No	124	99	1	1
Duration of illness (years)	< 5	28	15	1	1
	5–10	45	10	0.42 (0.16, 1.05)	0.48 (0.15, 1.54)
	< 10	107	190	3.32 (1.69, 6.48)	3.16 (1.31, 7.64)**
Previous hospitalization	Yes	48	104	2.58 (1.68, 3.94)	1.28 (0.66, 2.49)
	No	132	111	1	1

* p-value significant at p < 0.05, ** p-value is significant at p < 0.01, *** p-value is significant at p < 0.001 and p-value from Hosmer and Lemeshow test was 0.745

### Discussion

The prevalence of a current study is consistent with a study reported a meta-analysis in the USA 58% to acutely depressed and remitted patients [37]. Half of the Asian patients with MDD had all domains of cognitive dysfunction by assessing perceived deficit questionnaire-depression [14].

On the other hand, the prevalence of the current study is lower than studies done in British 83% [38] and USA 76.6% [24]. The variation of the prevalence might be due to used different study design, sample size difference, socio-cultural differences between those countries and Ethiopia, and measurement tools variation. In the USA cohort study design was used by assessing the psychiatric diagnostic screening questionnaires and in British was used systematic review study.

In addition to the above variation, the study time, at which the current data was collected, could be considered as a source of variation.

However, the current study finding was higher than a study carried out in Canada, in which the prevalence were reported to be 44% [39] and China 12.6% [40]. The possible reason could be different in measurement tools, study design, and age. In Canada, cognitive impairment was conducted by using prospective study design and it was screened by using Composite International Diagnostic Interview tool for 3-year residual symptoms and in China elderly people age greater than sixty. Another variation might be due to a year of study and the socio-cultural difference might account for the difference.

Regarding associated factors, the risk of CI for patients who did not read and write was increased by five times as compared to patients who achieved level of Education College and above. This is supported with other similar study conducted a meta-analysis of cognitive deficits in first-episode MDD were lower level of educational status the risk of severe deficits in cognitive functioning [21] and having a low level of education among elderly people of China [40].

Participants whose age late adulthood was three times exposed CI as compared to that of younger adults. It might be due to natural processing of age, they are vulnerable to medical and psychiatric co-morbidity and they took treatment for a longer period of time might contribute to cognitive impairment. Which was supported other studies done in Asian elderly people who had an age range between 21 and 40 years was highly cognitive impairment than 41–60 years old and in Egypt age one of the factors for cognitive dysfunction [14, 19].

Respondents who were not working were 3.63 times more likely to develop CI than those who were working which was consistent with studies conducted among Canada and British MDD patients. One-quarter the impact of MDD on work loss and low work performance was directly attributable to self-reported cognitive complaints and all depressed patients had the negative consequence of their work functioning [38, 41].

Participants having history of relapse were three times risk of to develop CI as compared to participants who did not have relapse, this was supported by studies were done with CI and residual symptoms among MDD patients were associated with higher rates of relapse and recurrence due to CI [26, 39, 42–44]. The recurrent episode or relapse of depression leads to more impaired their CI than a single episode of depressed patients [26] and in France multiple episodes of depression leads cognitive dysfunction [17].

Having co-morbid illness had two times more likely to develop CI as compared with those who had not co-morbidity among participants who had MDD with a psychotic feature. This is consistent with a study conducted in Denmark. The presence of co-morbid disorders had been shown to be a predictor of poor cognitive performance in depression which leads to cognitive dysfunction among depressed patients [45]. Participants who had a longer duration of illness 3.2 times to experienced CI as compared to patients who had with short duration illness. This is also supported by a study conducted in the University of Texas and Columbia to treatment resistance contributes to longer episodes [46] and another study in Chicago longer duration of illness was worsen the cognitive dysfunction [8].

### Conclusion

This study revealed that the prevalence of CI among major depressive disorder patients was high. Having relapses, being in late adulthood, inability to read and write, not currently working, long-term duration of illness and having co-morbid psychiatric illness were significantly associated with CI. Further research on risk factors for CI should be conducted to strengthen and broaden these findings.

## Limitation

This is a cross-sectional study; it is difficult to determine the temporal relationship between explanatory and outcome variables. This finding likely only hints at the complex interactions between CI and explanatory variables. Another most important limitation of this study is the MMSE scale was not validated in Ethiopia although it is widely used in USA and European countries as a screening tool of CI for geriatric patients but culture, education and age alterations in low income country like Ethiopia was considered.

## Additional file

**Additional file 1.** Distributions of substance-related factors among MDD patients at AMSH, Addis Ababa, Ethiopia 2017 (n = 395).

## Abbreviations

AD: antidepressant
AMSH: Amanuel Mental Specialized Hospital
CI: cognitive impairment
DSM: Diagnostic Statistical Manual of four and five
MDD: major depressive disorders
UOG: University of Gondar
WHO: World Health Organization
