# Supplementary material for: Magnitude and correlates of cognitive impairment among major depressive disorder patients in Addis Ababa: institution based cross-sectional study
Source: BMC Res Notes. 2019 Mar 14;12:135. doi: 10.1186/s13104-019-4184-5 (PMC6416877; doi:10.1186/s13104-019-4184-5)
Supplement: Supplementary file 1 — Additional file 1. Distributions of substance-related factors among MDD patients at AMSH, Addis Ababa, Ethiopia 2017 (n = 395). [file 13104_2019_4184_MOESM1_ESM.docx]

Additional file 1. Distributions of Substance-related factors among MDD patients at AMSH, Addis Ababa, Ethiopia 2017(n=395).

| **Variables** | **Frequency** | **Percent** |
| --- | --- | --- |
| **Lifetime substance use** |  |  |
| Yes | 126 | 31.9 |
| No | 269 | 68.1 |
| **Tobacco** |  |  |
| Yes | 69 | 17.5 |
| No | 326 | 82.5 |
| **Alcohol** |  |  |
| Yes | 67 | 17.0 |
| No | 328 | 83.0 |
| **Khat** |  |  |
| Yes | 97 | 24.6 |
| No | 228 | 75.4 |
| **Current substance use** |  |  |
| Yes | 114 | 28.9 |
| No | 281 | 71.1 |
| T**obacco** |  |  |
| Yes | 68 | 17.2 |
| No | 327 | 82.8 |
| **Alcohol** |  |  |
| Yes | 54 | 13.7 |
| No | 341 | 86.3 |
| **Khat** |  |  |
| Yes | 88 | 22.3 |
| No | 307 | 77.7 |
